# Polydatin Relieves Airway Remodeling by Inhibiting P2X7R–NLRP3‐Mediated Excessive Autophagy in Asthma

**DOI:** 10.1002/iid3.70331

**Published:** 2026-04-05

**Authors:** Guangxing Li, Liangchang Li, Zhiguang Wang, Yihua Piao, Yilan Song, Li Li, Chang Xu, Xiaowan Li, Guanghai Yan

**Affiliations:** ^1^ Jilin Key Laboratory for Immune and Targeting Research on Common Allergic Diseases Yanbian University Yanji Jilin China; ^2^ Department of Anatomy, Histology and Embryology Yanbian University Medical College Yanji Jilin China; ^3^ Department of Respiratory Medicine Affiliated Hospital of Yanbian University Yanji Jilin China; ^4^ Department of Intensive Care Unit Affiliated Hospital of Yanbian University Yanji Jilin China

**Keywords:** airway remodeling, asthma, autophagy, NLRP3, P2X7R, polydatin

## Abstract

**Background:**

Asthma is characterized by chronic airway inflammation and remodeling. Polydatin (PD), a natural compound, has shown anti‐inflammatory potential, but its role in asthma‐related airway remodeling and the underlying mechanisms involving the P2X7R–NLRP3 inflammasome axis and autophagy remain unclear.

**Methods:**

An ovalbumin‐induced asthmatic mouse model and primary airway smooth muscle cells (ASMCs) were used. Mice were treated with PD or the P2X7R agonist BzATP. Assessments included airway hyperresponsiveness, histopathology, inflammatory cell counts, cytokine profiling (ELISA), flow cytometry for T‐cell subsets, and protein analysis via western blot and immunofluorescence. Autophagy was evaluated by measuring LC3‐I/II, Beclin‐1, and acidic vesicular organelles. Key signaling molecules (P2X7R, NLRP3, ASC, caspase‐1, LKB1/AMPK/mTOR) were analyzed. In vitro, ASMCs were treated with BzATP, PD, and specific inhibitors; P2X7R was silenced using siRNA.

**Results:**

PD treatment significantly alleviated ovalbumin‐induced airway hyperresponsiveness, inflammatory cell infiltration, goblet cell hyperplasia, and collagen deposition in mice. It restored the Th1/Th2 and Th17/Treg balance and reduced levels of airway remodeling markers (α‐SMA, PCNA, VEGF, MMP‐9). PD suppressed excessive autophagy (reduced LC3‐I/II and Beclin‐1) and modulated the LKB1/AMPK/mTOR pathway. Furthermore, PD inhibited the ATP/P2X7R axis, leading to reduced NLRP3 inflammasome activation, caspase‐1 activity, and secretion of IL‐1β and IL‐18. In ASMCs, PD reversed BzATP‐induced autophagy and NLRP3 activation. Silencing P2X7R enhanced AMPK phosphorylation, underscoring its role in the pathway.

**Conclusion:**

PD attenuates airway remodeling in asthma by inhibiting the ATP/P2X7R–NLRP3 inflammasome axis and subsequent excessive autophagy, partly through modulation of the LKB1/AMPK/mTOR signaling pathway. These findings highlight PD as a promising therapeutic candidate for asthma treatment.

## 1 Introduction

Asthma has been characterized by chronic inflammatory changes, airway hyperresponsiveness, and severe airway remodeling [1]. Th1/Th2 imbalance is considered to be the main mechanism of asthma [2, 3], but recent studies [4, 5] have also shown that Th17/Treg has an important role in allergic diseases. The incidence and mortality of asthma are increasing year by year. The pathogenesis of asthma is rather complex [6]. The changes in the airway caused by the reversible and/or irreversible obstruction would eventually lead to abnormal changes in airway structure [7]. Airway smooth muscle cell (ASMC) proliferation plays an important role in airway remodeling [8]. Therefore, the abnormal proliferation of ASMCs and the possible signal transduction mechanism underlying the airway remodeling should be further studied.

Autophagy is a highly conserved process of cell self‐digestion and dissolution mediated by lysosomes only in eukaryotic cells. During autophagy, the organelles to be degraded would form a double‐membrane autophagosome, which is then degraded in the lysosome [9]. The autophagic process is rather complicated and needs not only stimuli but also the co‐regulation of related genes and proteins [10]. However, prolonged autophagy development, as well as excessive autophagy, can disrupt the intracellular environment and homeostasis. This disruption not only amplifies the inflammatory response but also facilitates autophagic cellular apoptosis [11]. Accumulating evidence has confirmed the role of autophagy in asthma [12, 13]. In the studies concerning autophagy and LKB1/AMPK/mTOR signaling pathway, mTOR has been considered to be the inhibiting signal for autophagy [14, 15]. Activation of AMPK negatively regulates the mTOR activity, suggesting its role in asthma prevention and treatment [16]. However, autophagy is not entirely dependent on the mTOR pathway in inflammatory diseases [17, 18]. Thus, it is necessary to illustrate the role and mechanism of autophagy in airway remodeling of asthma.

ATP is an important physiological activator of P2X7R. High concentrations of extracellular ATP would activate P2X7R, and the P2X7 overexpression mediates the NLRP3 activation [19]. There are three key components, that is, caspase‐1, ASC, and NLRP3, in the NLRP3 inflammasome [20]. NLRP3 activation and autophagy can regulate each other in inflammation‐related diseases [21]. Noteworthy, the P2X7R–NLRP3 axis is involved in asthma [22, 23].

**Figure 1:**
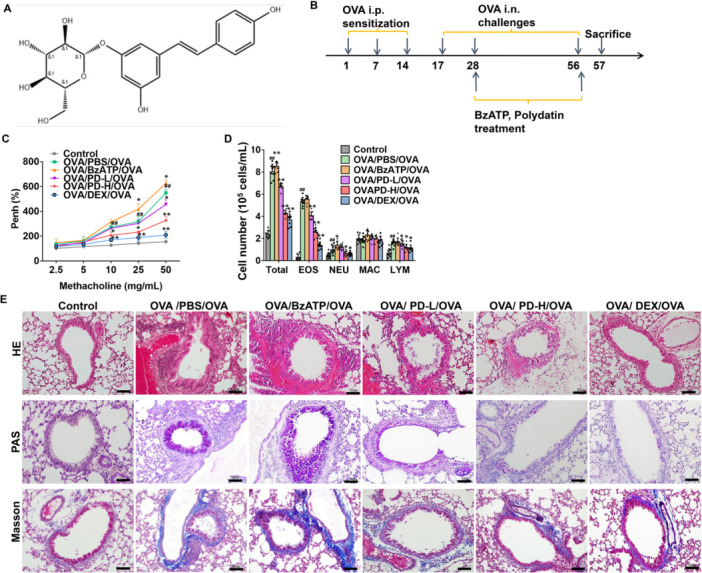
Effects of PD on airway hyperresponsiveness and inflammation in OVA‐inhaled mice. (A) The chemical structure of PD. (B) A schematic diagram of the experimental protocol. (C) Airway hyperresponsiveness, expressed as Penh (%), was measured at 24 h after the last challenge. (D) The numbers of total and individual cell types in bronchoalveolar lavage fluid were counted. EOS, eosinophil; NEU, neutrophil; MAC, macrophage; LYM, lymphocyte. (E) Lung sections were stained with hematoxylin–eosin (HE), periodic acid‐Schiff (PAS), and Masson's trichrome. Scale bar, 50 μm. ^##^ *p* < 0.01 versus Control; *p < 0.05, **p < 0.01 versus OVA/PBS/OVA.

At present, corticosteroids remain an effective asthma treatment. For airway remodeling, even high‐dose corticosteroids have a poor effect. Additionally, glucocorticoids have side effects. Natural herbal medicines and their components have shown distinct advantages in the management of asthma and its complications. Polydatin (PD; C_20_H_22_O_8_; chemical structure in Figure 1A) is the main active ingredient of the rhizome of *Polygonum cuspidatum* [24]. PD is also found in peanuts, grapes, red wine, and cocoa products. It may exert anti‐inflammatory and antioxidant effects, and improve myocardial ischemia and reperfusion [25, 26, 27, 28]. In particular, PD can alleviate asthma [29]. PD can attenuate nonalcoholic steatohepatitis by inhibiting mTOR signaling and upregulating TFEB expression and activity [30]. Interestingly, one study has shown that PD enhanced autophagy flux in cardiac dysfunction by upregulating Sirt3 in diabetic mice [31]. However, another study reports that PD protects β‐cells from lipotoxicity‐induced type 2 diabetes mellitus by inhibiting excessive autophagy [11]. This suggests that PD exerts different effects on autophagy induced by different molecular mechanisms in similar diseases. Up to now, the effects and mechanisms of PD on autophagy in airway remodeling have not been reported. Studies found that PD inhibits NLRP3 inflammasome in dry eye disease [32] and nonsmall cell lung cancer [33]. P2X7R has been confirmed to be the upstream signal of NLRP3. Moreover, P2X7R could regulate autophagy in many diseases [34, 35]. However, whether PD can regulate autophagy in asthma and the underlying mechanism remains unclear.

Herein, the effects of PD on airway remodeling were investigated. Notably, our research introduces a novel perspective by focusing on the regulatory effects of PD on the ATP/P2X7R–NLRP3 inflammasome axis and LKB1/AMPK/mTOR autophagy signaling pathway in airway remodeling. Our findings may provide a new research direction for asthma and autophagy, and provide evidence for asthma treatment.

## 2 Materials and Methods

### 2.1 Study Animals

BALB/C mice (female; 6–8‐week‐old; weight 20 ± 2 g, *n* = 48; and 4–6‐week‐old, weight 18–20 g, *n* = 20) were from the experimental animal center of Yanbian University (Yanbian, Jilin, China). The license number of the laboratory animal was SYXK2020‐0009. The experimental procedures were approved by the Ethics Committee of the Medical College of Yanbian University (Approval Number: JN.No20190515b068). The work described has been carried out in accordance with the relevant guidelines and regulations. All animal studies complied with the ARRIVE guidelines.

### 2.2 Animal Treatment and Grouping

Six groups (*n* = 8) were set up, that is, the control, OVA/PBS/OVA, OVA/BzATP/OVA, OVA/PD‐L/OVA (OVA + low dose of PD), OVA/PD‐H/OVA (OVA + high dose of PD), and OVA/DEX/OVA (OVA + dexamethasone) groups. The experimental procedures of asthma animal model establishment were shown in Figure 1B. On Days 1, 7, and 14, the mice in the OVA/PBS/OVA, OVA/BzATP/OVA, OVA/PD‐L/OVA, OVA/PD‐H/OVA, and OVA/DEX/OVA groups received an intraperitoneal injection (i.p.) of 50 μg ovalbumin (OVA; Sigma, St. Louis, MO, USA) in 0.2 mL saline and 4 mg aluminum hydroxide. The mice in the OVA/BzATP/OVA group were administered i.p. with 50 mg/kg BzATP (ab120444; purity > 94%; Abcam) every day for 4 weeks, starting on Week 4. The mice in the OVA/PD‐L/OVA and OVA/PD‐H/OVA groups were administered i.p. with PD (20 mg/kg or 40 mg/kg; purity > 98%; TW Reagent Co. Ltd, Shanghai, China) three times a week for 4 weeks, with each dose given 30 min before the OVA inhalation challenge. The dosage of PD was determined based on previous studies [36, 37, 38] and our pilot experiments. The OVA/DEX/OVA group was given i.p. DEX (Sigma). Additionally, from Day 17, an airway challenge was conducted (3 times a week, 30 min each time) through aerosolizing 2% OVA for 4 weeks. An equal amount of normal saline was given in the control group. The airway hyperresponsiveness was measured at 24 h after the last administration. Then, animals were sacrificed. Lung tissues and BALF (bronchoalveolar lavage fluid) were collected for subsequent analysis.

### 2.3 ASMCs Isolation, Culture, and Treatment

Primary ASMCs were isolated from the trachea and proximal bronchi of 4–6‐week‐old BALB/c mice, as previously described [39]. Briefly, tissues were minced, digested with collagenase II (1.5 mg/mL) and elastase (0.5 mg/mL), and purified via centrifugation. ASMCs were characterized by immunofluorescence for calponin and smooth muscle myosin heavy chain expression, spindle‐shaped morphology, and carbachol‐induced contraction. They were cultured in DMEM/F12 medium supplemented with 10% fetal bovine serum, 1% insulin–transferrin–selenium, and 0.1% 100× penicillin–streptomycin at 37°C/5% CO_2_. Experiments used cells at passages 3–5.

ASMCs were stimulated with BzATP (100 μM) for 24 h. Before BzATP stimulation, these cells were preincubated with Apyrase (10 U/mL; Sigma) for 30 min, 10 mM 3‐MA for 4 h, 10 μM A438079 (Abcam, Cambridge, UK) for 1 h, 10 μM caspase‐1 inhibitor (Sigma) for 1 h, 20 or 40 μM PD for 4 h, and 100 μM Z‐YVAD‐FMK (Abcam) for 4 h. Thereafter, the ASMCs and culture supernatant were collected for the subsequent analyses.

### 2.4 MTT Assay

ASMCs were plated onto 96‐well plates (5 × 10^4^ cells/mL). The blank wells and PBS wells were set up. Before the assessment, the cells were incubated with PD (0, 1, 10, 50, and 100 μM, respectively). After discarding the culture medium, 20 μL MTT (5 mg/mL) was added for incubation in the dark for 4 h, followed by incubation with 200 μL DMSO at 37°C for 10 min. The OD value at 490 nm was detected, and the cell viabilities were calculated accordingly.

### 2.5 Assessment of Airway Hyperresponsiveness

The animals were anesthetized with pentobarbital sodium (100 mg/kg) at 24 h after the last challenge. The mouse tracheas were cannulated and ventilated at 3.0 cm H_2_O positive end‐expiratory pressure and 150 breaths/min. Subsequently, the aerosolized methacholine challenge (at concentrations of 3.125, 6.25, 12.5, and 25 mg/mL) was conducted. After that, airway hyperresponsiveness was quantitatively assessed using noninvasive whole‐body plethysmography (WBP‐4MR, Tow‐int Tech, Shanghai, China) and expressed as the Penh index, which was calculated from pressure waveform deviations during expiration and reported as the average of the three highest values following each dose.

### 2.6 BALF Collection and Cell Count

After ethyl ether anesthesia, BALF collection was conducted by lavaging the lungs with PBS (0.8 mL). After centrifugation, the supernatant BALF sample was collected. Diff‐Quik staining (International Reagents, Kobe, Japan) was conducted on cell pellets. The total and differential cell counts were measured by each cell type with a hemocytometer.

### 2.7 Lung Histopathology

The left lungs were made into 5‐µm sections. Staining with Masson's trichrome, periodic acid‐Schiff (PAS), and hematoxylin–eosin (H&E) was performed. At least four sections per lung tissue sample were evaluated.

### 2.8 ELISA

The levels of interleukin 4 (IL‐4), IL‐5, IL‐13, IL‐17, IL‐22, interferon‐γ (IFN‐γ), IL‐1β and IL‐18 were measured with ELISA Kits (R&D Systems). The sensitivities for IL‐4 and IFN‐γ were 2.0 pg/mL. The sensitivities for IL‐5, IL‐13, IL‐17, IL‐22, IL‐1β, and IL‐18 were 7.0, 1.5, 5.0, 5.8, 4.8, and 25 pg/mL, respectively.

### 2.9 Western Blot Analysis

After extraction from the right lung tissues and ASMCs, proteins were separated and transferred onto the membrane. After blocking for 2 h, the membrane was probed with primary antibodies at 4°C overnight, and subsequently with corresponding secondary antibodies at 37°C for 1 h. The primary antibodies were as follows: α‐SMA (#19245; Cell Signaling Technology (CST)), PCNA (ab29; Abcam), VEGF (ab51874; Abcam), MMP‐9 (ab38898; Abcam), LC3‐I/II (ABC929; Sigma), Beclin‐1 (ab62557; Abcam), LKB1 (Ser428; #3482; CST), LKB1 (#3047; CST), AMPKα (Thr172; #2535; CST), AMPKα (#5831; CST), mTOR (Ser2448; #2971; CST), mTOR (#2983; CST), P2X7R (77665; Thermo), NLRP3 (MA523919; Lifescience), ASC (#67824; CST), Caspase‐1 (ab1872; Abcam), IL‐1β (#12703; CST), cleaved‐IL‐1β (#83186; CST), pro‐IL‐18 (M156‐3; MBL BEIJING BIOTECH), cleaved‐IL‐18 (M157‐3; MBL BEIJING BIOTECH) and β‐actin (#3700; CST). The secondary antibodies included the goat anti‐mouse IgG HRP (ab205719; Abcam), goat anti‐rat IgG‐HRP (ab205720; Abcam), and goat anti‐rabbit IgG HRP (ab205718; Abcam). Protein blots were visualized by the ImageJ software (version 1.53a, National Institutes of Health, USA).

### 2.10 Flow Cytometry

Single‐cell suspension from lung tissues was incubated with PE‐cy7‐CD4 and FITC‐CD3e. After fixation and permeabilization, intracellular staining of CD4+ Th cells was conducted with FITC‐IFN‐γ, FITC‐IL‐4, PE‐IL‐17A, and FITC‐FoxP3. Then, all samples were measured on a BD CytoFLEX flow cytometer and analyzed with FlowJo. All antibodies were from eBioscience (San Diego, CA, USA).

### 2.11 Immunohistochemistry

Immunostaining was performed with anti‐α‐SAM (#19245; CST), anti‐PCNA (ab29; Abcam), anti‐VEGF (ab51874; Abcam), and anti‐MMP‐9 (ab38898; Abcam) primary antibodies, and then with secondary antibodies. After hematoxylin counterstaining, the sections were observed. The positive area of each protein was analyzed using ImageJ software.

### 2.12 Immunofluorescence

ASMCs were planted in the 6‐well chamber and treated accordingly. Then, the cells or lung tissue in each group were fixed and permeabilized. After blocking, incubation with the anti‐P2X7R (77665; Thermo) and anti‐LC3B (ab43894; Abcam) primary antibodies at 4°C overnight and with secondary antibody (Invitrogen) for 2 h was conducted. Fluorescence was analyzed under Cytation 5 microscope (BioTek, USA). The relative fluorescent intensity of each protein was evaluated using ImageJ software.

Acid vesicular organelles (AVOs) were analyzed by acridine orange dye staining. The dye emitted green fluorescence in the nucleus and cytoplasm, and red fluorescence in the formed AVOs. ASMCs were plated in a 6‐well plate and stained with AO for 15 min. Images were acquired with Cytation 5 microscope (BioTek).

### 2.13 RNA Interference

P2X7R siRNA (AMBION, Thermo Fisher Scientific, USA) was transfected into ASMCs with Lipofectamine 3000 (Invitrogen). Silencing efficiency was assessed with RT‐PCR at 72 h of transfection.

### 2.14 Assessment of Caspase‐1 Activation

Caspase‐1 activity was measured with colorimetry (Beyotime, Jiangsu, China). Total protein extract (20 μg) was incubated with the catalytic substrate of caspase‐1, ace‐tyl‐Tyr‐Val‐Ala‐Asp *p*‐nitroaniline, at 37°C for 2 h. The OD value at 405 nm was measured on a microplate reader (BioTek). Caspase‐1 activity was represented by the yellow chromophore.

### 2.15 Statistical Analysis

Data are expressed as mean ± SEM and were assessed with GraphPad Prism 7. For multiple comparisons, ANOVA and Dunnett's post hoc test were conducted. The *t‐*test compared the differences between the two groups. *p* < 0.05 indicates a significant difference.

## 3 Results

### 3.1 PD Treatment Attenuates Inflammation in Asthma Mice

Airway hyperresponsiveness in asthma mice was measured after aerosolized methacholine challenge. Compared to the Control group, the OVA/PBS/OVA group had significantly higher airway resistance, represented by Penh% (Figure 1C). Moreover, the airway resistance of the BzATP‐treated group increased significantly than the OVA/PBS/OVA group. Furthermore, the airway resistance of mice treated with low‐ and high‐dose PD was significantly decreased than the OVA/PBS/OVA group. These results suggest that PD alleviates airway hyperresponsiveness in asthma mice.

Then, the immune cells in BALF were detected. Compared to the OVA/PBS/OVA group, the number of total cells, neutrophils, and lymphocytes in BALF of the BzATP group increased (Figure 1D), indicating that BzATP could aggravate the airway remodeling of asthmatic mice. On the other hand, the number of eosinophils, neutrophils, lymphocytes, and total cells in the high‐dose PD group was significantly decreased, indicating that PD could reduce the number of inflammatory cells in the BALF of asthmatic mice.

As revealed by HE staining, more severe pathological changes were observed in the OVA/PBS/OVA group than Control group (Figure 1E). Pathological changes were alleviated in the PD groups but were aggravated in the BzATP group. Moreover, the PAS staining revealed hyperplasia and mucus secretions in the OVA and BzATP groups. However, such effects were reduced in the low‐ and high‐dose PD groups. Furthermore, the Masson staining showed smooth muscle hyperplasia in the OVA and BzATP groups. The smooth muscle hyperplasia was alleviated in the low‐ and high‐dose PD groups. Collectively, PD significantly attenuated the effects of BzATP by reducing the inflammatory cells and inhibiting goblet cell proliferation and collagen deposition.

### 3.2 PD Alleviates the Imbalance of Th1/Th2 and Th17/Treg in Asthma Mice

**Figure 2:**
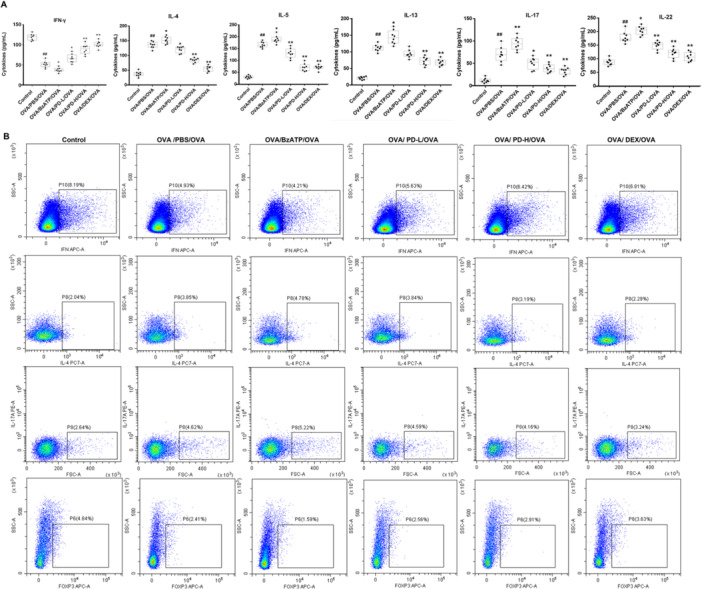
Effects of PD on the different subsets of Th cells. (A) The inflammatory factor levels of IFN‐γ, IL‐4, IL‐5, IL‐13, IL‐17, and IL‐22 in the bronchoalveolar lavage fluid of asthma mice were quantified by ELISA. (B) Flow cytometry analysis of cells. CD4^+^ cells were gated for analyzing IFN‐γ, IL‐4, IL‐17, and Foxp3. ^##^ *p* < 0.01 versus Control; *p < 0.05, **p < 0.01 versus OVA/PBS/OVA.

Cytokine balance in Th1/Th2 and Th17/Treg cells exerts a vital role in allergic diseases [40], and the IL‐22 increases in asthmatic mice [41]. To elucidate the effects of PD on the presence of effector T cells, the cytokines in BALF were measured with ELISA. Compared to the control, IL‐4, IL‐13, IL‐5, IL‐22, and IL‐17 levels in BALF were increased, while IFN‐γ was decreased in BALF of the OVA group (Figure 2A), which was significantly reversed by PD. Nevertheless, BzATP worsened the imbalance of cytokines induced by asthma. Flow cytometry showed that, in asthmatic mice, there were higher percentages of IL‐4^+^ cells (Th2 cells) and IL‐17^+^ cells (Th17 cells) (Figure 2B). However, they were dramatically decreased with the administration of high‐dose PD but increased with the administration of BzATP. The percentages of IFN‐γ^+^ cells (Th1 cells) and Foxp3^+^ cells (Treg cells) were suppressed when compared to Control mice. BzATP enhanced the changes, but PD treatment markedly inhibited these changes. To sum up, these results suggested that PD maintained the balance of Th1/Th2 and Treg/Th17.

### 3.3 PD Prevents Airway Remodeling in Asthma Mice

**Figure 3:**
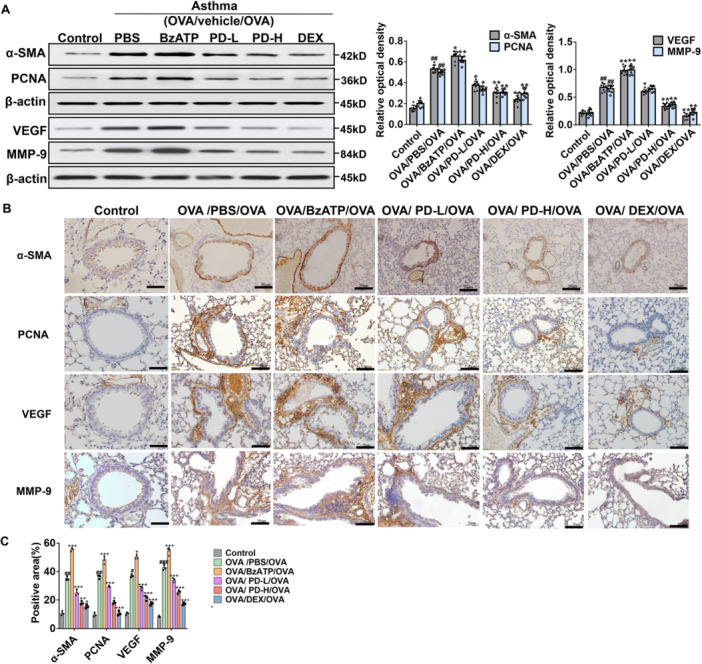
Effects of PD on expressions of α‐SMA, PCNA, VEGF, and MMP‐9 in lung tissues of OVA‐inhaled mice. (A) The protein expression levels of α‐SMA, PCNA, VEGF, and MMP‐9 were analyzed by western blot analysis. (B) Immunohistochemistry was performed to assess the distribution of α‐SMA, PCNA, VEGF, and MMP‐9. Scale bar, 50 μm. (C) The positive area (%) of each protein is presented. ^#^ *p* < 0.05, ^##^ *p* < 0.01 versus Control; *p < 0.05, **p < 0.01, ***p < 0.001 versus OVA/PBS/OVA.

There are also basement membrane thickening, neovascularization, and ASMC proliferation in airway remodeling [42]. Herein, western blot revealed that α‐SMA, PCNA, VEGF, and MMP‐9 levels were elevated in the OVA/PBS/OVA group than the Control group (Figure 3A). However, their levels were decreased in the high‐dose PD groups, while increased in the BzATP group. Immunohistochemistry analysis revealed that the PD decreased α‐SMA, PCNA, VEGF, and MMP‐9 protein levels in the lung tissues of asthmatic mice (Figure 3B), with significantly reduced positive area of each protein in the low‐dose and high‐dose PD groups (Figure 3C). These results indicate that PD reduces matrix deposition and angiogenesis in the lung tissue of asthma mice.

### 3.4 PD Decreases Excessive Autophagy and Regulates LKB1/AMPK/mTOR Signaling in Asthmatic Mice

**Figure 4:**
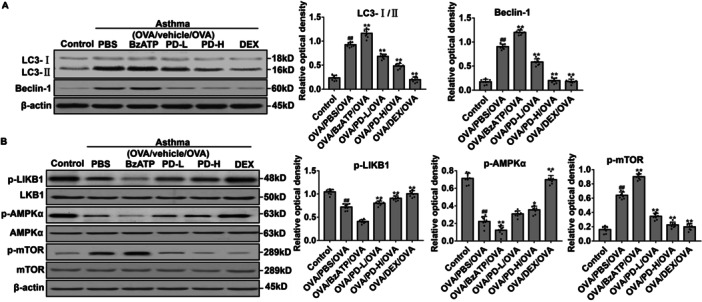
Effects of PD on autophagy and LKB1/AMPK/mTOR signaling pathway in lung tissues of OVA‐inhaled mice. Protein expression was analyzed using western blot. (A) The representative and quantitative western blot results of LC3‐I/II and Beclin‐1. (B) The representative and quantitative western blot results of LKB1, p‐LKB1, AMPK, p‐AMPK, mTOR, and p‐mTOR. ^##^ *p* < 0.01 versus Control; *p < 0.05, **p < 0.01 versus OVA/PBS/OVA.

The effects of PD on autophagy in asthmatic mice were next explored. Western blot demonstrated that, in the OVA/PBS/OVA group and BzATP‐treated group, LC3‐I/II and Beclin‐1 levels increased. However, PD reversed the changes of these proteins in asthmatic mice (Figure 4A). LKB1, AMPK, and mTOR protein levels were then measured. The phosphorylation and expression levels of LKB1 and AMPK were downregulated, while those of mTOR were upregulated in the OVA/PBS/OVA group than the Control group (Figure 4B). Moreover, the phosphorylation levels of LKB1, AMPK, and mTOR were also affected by BzATP and PD treatments. PD upregulated LKB1 and AMPK phosphorylation while reducing mTOR phosphorylation. These results suggest that PD may inhibit excessive autophagy and regulate the LKB1/AMPK/mTOR signaling in asthmatic mice.

### 3.5 PD Activates ATP/P2X7 Axis‐Mediated NLRP3 Inflammasome of Asthmatic Mice

**Figure 5:**
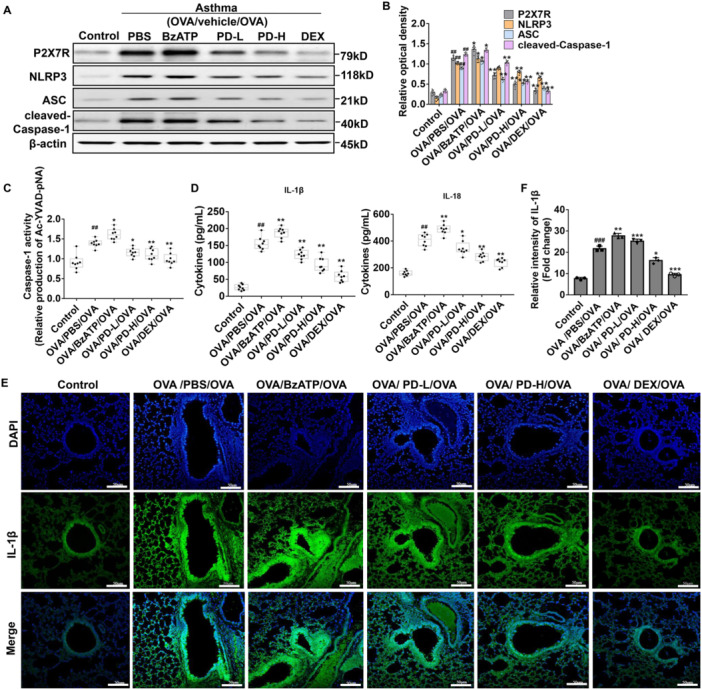
Effects of PD on P2X7R and NLRP3 inflammasome in OVA‐inhaled mice. (A) The levels of P2X7R, NLRP3, ASC, and caspase‐1 in lung tissues were measured with western blot analysis. (B) Quantitative western blot results. (C) The caspase‐1 activity in BALF supernatant from each group was detected by colorimetry. (D) The levels of IL‐1β and IL‐18 in BALF supernatant from each group were detected by ELISA. (E) Immunofluorescence staining of IL‐1β (green) in lung tissues (nuclei, blue). Scale bar, 50 μm. (F) Statistical results of the relative intensity of IL‐1β (expressed as fold change). ^##^ *p* < 0.01, ^###^ *p* < 0.001 versus Control; *p < 0.05, **p < 0.01, ***p < 0.001 versus OVA/PBS/OVA.

The involvement of the ATP/P2X7 axis and NLRP3 inflammasome in asthma was investigated. Western blot revealed that the BzATP up‐regulated P2X7R, NLRP3, ASC, and caspase‐1, while low‐ and high‐dose treatments of PD reduced their levels compared to the OVA/PBS/OVA group (Figure 5A,B). As presented in Figure 5C–F, the OVA/PBS/OVA group had elevated caspase‐1 activity and IL‐1β and IL‐18 levels than the Control group. They were further increased by BzATP, whereas inhibited by PD. Taken together, PD inhibits ATP/P2X7 activation and NLRP3 inflammasome.

### 3.6 Effects of PD on BzATP‐Induced Excessive Autophagy and Its Regulation of LKB1/AMPK/mTOR Signaling in ASMCs

**Figure 6:**
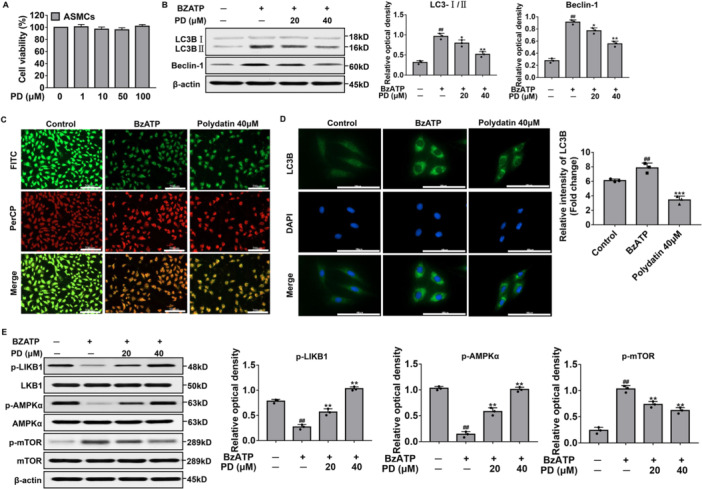
Effects of PD on autophagy and LKB1/AMPK/mTOR in BzATP‐treated ASMCs. (A) Cell viability detected by MTT assay. (B) The protein expression levels of LC3‐I/II and Beclin‐1 were analyzed by western blot analysis. (C) Detection and quantification of AVOs in BzATP‐treated ASMCs by immunofluorescence. Scale bar, 200 μm. FITC (green) showed cytoplasm and nucleus, and PerCP (red) showed AVOs. (D) The immunofluorescence analysis of LC3B (green) in ASMCs. Scale bar, 100 μm. The relative intensity of LC3B (expressed as fold change) is also shown. (E) LKB1, AMPK, mTOR, and phosphorylation of LKB1, AMPK, and mTOR were analyzed by western blot analysis. ^##^ *p* < 0.01, versus Control; *p < 0.05, **p < 0.01, ***p < 0.001 versus BzATP‐treated group.

The cell viability of ASMCs after 24 h of PD treatment was assessed with the MTT assay. ASMCs were incubated with indicated concentrations of PD from 1 to 100 μM. PD had no significant effects on the viability of ASMCs (*p* > 0.05) (Figure 6A). Concerning the effects of PD on the autophagy of ASMCs induced by BzATP, Western blot demonstrated that the BzATP treatment significantly elevated LC3‐I/II and Beclin‐1, while the low‐dose PD reduced their levels, and more significant effects were observed for the high‐dose PD treatment (Figure 6B). Moreover, the ASMCs stimulated by BzATP were stained with AO dye to evaluate the formation of AVOs. Our results from immunofluorescence showed that the BzATP treatment induced an increase in AVO formation in ASMCs, and the PD treatment reduced the AVOs in BzATP‐treated ASMCs (Figure 6C). Nevertheless, immunofluorescence results showed that BzATP markedly increased LC3B expression and PD pretreatment suppressed LC3B expression (Figure 6D). To demonstrate the effects of PD on the LKB1/AMPK/mTOR signaling pathway in BzATP‐induced ASMCs, the phosphorylation levels of LKB1/AMPK/mTOR signal were detected with western blot. As shown in Figure 6E, the phosphorylation levels of LKB1 and AMPK were downregulated, while those of mTOR were upregulated. Moreover, the PD treatment inhibited the effects of BzATP on the LKB1/AMPK/mTOR signaling pathway.

### 3.7 Effects of PD on ATP/P2X7R Axis‐Activated NLRP3 Inflammasome in ASMCs

**Figure 7:**
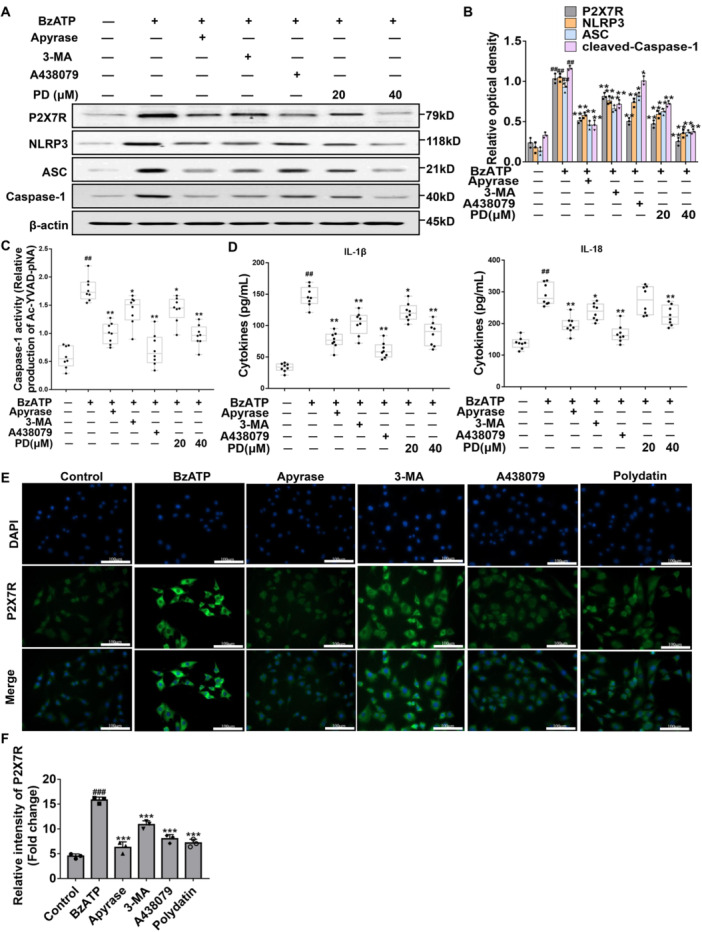
Effects of PD on P2X7R and NLRP3 inflammasome in BzATP‐induced ASMCs. (A) Western blot analyses of P2X7R, NLRP3, ASC, and caspase‐1 in response to the treatment with Apyrase, 3‐MA, A428079, or different doses of PD in ASMCs. (B) Relative protein level. (C) The caspase‐1 activities in ASMCs administered with Apyrase, 3‐MA, A428079, or different doses of PD were detected by colorimetry. (D) The levels of IL‐1β and IL‐18 in ASMCs, which were administered with Apyrase, 3‐MA, A428079, or different doses of PD, were detected by ELISA. (E) The immunofluorescence analysis of P2X7R (green) in ASMCs. Scale bar, 100 μm. (F) The relative intensity of P2X7R (expressed as fold change) is also presented. ^##^ *p* < 0.01 versus control; *p < 0.05, **p < 0.01, ***p < 0.001 versus BzATP‐induced group.

The underlying mechanism of PD on the ATP/P2X7 axis was investigated in ASMCs. Our results showed that the P2X7R level and NLRP3 inflammasome were highly expressed after the BzATP treatment, while the P2X7R and NLRP3 inflammasome levels were lower after the treatments of Apyrase, 3‐MA, A438079, and PD, in comparison with BzATP (Figure 7A,B). Moreover, caspase‐1 activity, IL‐1β, and IL‐18 levels were increased following the BzATP administration, while the administrations of Apyrase, 3‐MA, A438079, and PD reduced those in the ASMCs (Figure 7C,D). Similarly, our results from immunofluorescence indicated that BzATP stimulated the P2X7R expression in ASMCs, while Apyrase, 3‐MA, A438079, and PD reduced the P2X7R expression in BzATP‐induced ASMCs (Figure 7E,F).

### 3.8 Production and Cleavage of IL‐1β and IL‐18 Initiated by BzATP Are Prevented by PD in ASMCs

**Figure 8:**
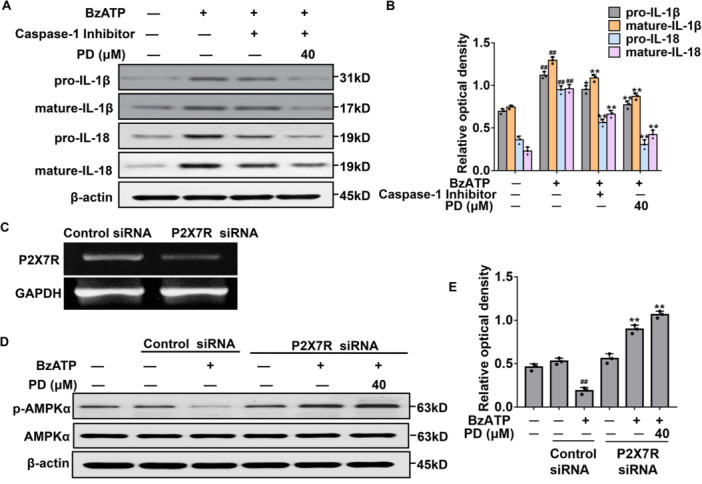
Effects of P2X7R low expression on AMPK in BzATP‐induced ASMCs. (A) Protein expression levels of pro‐IL‐1β, mature IL‐1β, pro‐IL‐18, and mature IL‐18 in ASMCs treated with caspase‐1 inhibitor, BzATP, or 40 μM PD were detected by western blot analysis. (B) Relative protein level. (C) The expressions of P2X7R in the P2X7R‐silenced ASMCs were verified by RT‐PCR. (D) The protein expression levels of p‐AMPK and AMPK in ASMCs were detected by western blot analysis after knockdown with P2X7R siRNA. (E) Relative protein level. ^##^ *p* < 0.01 versus control; *p < 0.05, **p < 0.01 versus BzATP‐induced group.

Whether PD interferes with BzATP‐stimulated IL‐1β and/or IL‐18 production was then investigated. Our results showed that the pro/mature‐IL‐1β and pro/mature‐IL‐18 expression were low in ASMCs, which was increased after stimulation with BzATP (Figure 8A,B). Moreover, pretreatment of PD before BzATP stimulation successfully decreased pro/mature‐IL‐1β and IL‐18. Furthermore, the caspase‐1 inhibitor IV also dramatically suppressed the production of pro/mature‐IL‐1β and IL‐18. Taken together, the inhibiting effects of PD on IL‐1β and IL‐18 production and cleavage functions may be mediated through P2X7R–NLRP3 inflammasome.

### 3.9 AMPK Activation Is Related to P2X7R Inhibition in ASMCs

Whether the P2X7R activation plays a role in AMPK‐dependent airway remodeling in ASMCs is explored. P2X7R was silenced in ASMCs with siRNA P2X7R (Figure 8C). The depletion of P2X7R induced the recovery of AMPK phosphorylation in BzATP‐stimulated ASMCs (Figure 8D,E). Interestingly, with the P2X7R deficiency, PD pretreatment further enhanced the AMPK phosphorylation level. Taken together, these results suggest that P2X7R inhibition by PD would cause alleviation of airway remodeling.

## 4 Discussion

Allergic asthma is a complex type of airway disease with various pathological changes [43]. Current treatment options can hardly reverse or cure airway remodeling, which might eventually lead to lung failure [44]. Thus, more effective drugs for the treatment of asthma are needed. The imbalance of Th1/Th2 and Th17/Treg plays important roles in allergic asthma [45]. As an endogenous risk factor, ATP would stimulate the selective expression of P2X7R in various cell types. ATP/P2X7 axis is involved in inflammatory response [19]. Moreover, BzATP is a specific activator of P2X7R [46]. Herein, ATP/P2X7 axis activation induced the secretion of inflammatory factors by Th2, Th17, and Th22. In addition, ATP/P2X7 axis activation by BzATP enhanced airway inflammation, mucus formation, fiber deposition, and airway hyperresponsiveness in lung tissues, while PD reversed these changes.

PD has traditionally been used as an antitussive, antiasthmatic, expectorant, and can regulate blood lipid levels [25, 26, 27, 28]. However, with the in‐depth study on its drug metabolism and pharmacology, PD has been found to have versatile roles and various beneficial functional effects, such as anti‐inflammation, scavenging free radicals, antioxidant, anti‐ischemic injury, liver protection, and antiapoptosis [26, 27, 28]. In our previous preliminary results, we found that PD inhibited the acute exacerbation of airway inflammation in asthmatic mice. Autophagy plays a role in asthma. However, the effects and mechanisms of PD on autophagy in airway remodeling have not been reported. Therefore, we explored whether PD participates in the remission of asthma through the regulation of autophagy.

Autophagy has been generally considered as a normal physiological process involved in homeostasis and normal cell survival in vivo [47]. However, in some extreme conditions (such as hypoxia, oxidative stress, insufficient nutrition, and ATP and other external factors), cellular autophagy will be more intense for self‐preservation [48, 49, 50]. Prolonged or excessive autophagy has been associated with disease progression through gene regulation and immune responses [51]. Among autophagy‐related proteins, LC3B (microtubule‐associated protein 1 light chain 3β) and Beclin‐1 (mammalian homolog of yeast Vps30/Atg6) are most widely studied [52]. Our results demonstrated that Beclin‐1 and LC3‐Ⅰ/LC3‐Ⅱ were upregulated in the OVA‐induced asthmatic airway remodeling mice. However, these effects were inhibited by PD.

Moreover, our results showed that the ATP/P2X7R axis enhanced the expressions of caspase‐1, ASC, and NLRP3 and activated the NLRP3 inflammasome as well as the subsequent formation of the ATP/P2X7R–NLRP3 axis. PD inhibited the expressions of ASC, caspase‐1, and NLRP3 by blocking the ATP/P2X7R axis. Interestingly, we revealed that Beclin‐1 and LC3‐Ⅰ/LC3‐Ⅱ were upregulated in the BzATP‐treated mice. It may suggest the relationship between the P2X7R signal and autophagy in the airway remodeling of asthma. P2X7R has been reported to promote the activation of autophagy in other diseases [51, 52]. In addition, activation of autophagy in asthma can lead to overactivation of the NLRP3 inflammasome, while inhibition of autophagy can alleviate asthma inflammation [53, 54]. These results were consistent with our findings. In a previous study concerning the classical autophagy pathways, autophagy was associated with the AMPK/mTOR pathway [14]. AMPK phosphorylation would inhibit the activity of mTOR, which plays an important role in autophagy, and the inhibition of mTOR would induce autophagy [55]. Moreover, LKB1 is the upstream signal pathway of AMPK. To determine whether autophagy is related to the classic autophagy pathway in asthmatic airway remodeling, the phosphorylation levels of the LKB1/AMPK/mTOR pathway were measured in this study. Our results showed that, in the OVA‐induced airway remodeling, the phosphorylation level of LKB1/AMPK was downregulated, while the phosphorylation level of mTOR was upregulated. The results indicate that autophagy might not be or hardly regulated by the mTOR signaling pathway, in line with previous findings that there are mTOR‐dependent and ‐independent pathways of autophagy [56, 57]. However, we found that although PD did not inhibit autophagy in asthma via LKB1/AMPK/mTOR pathway, PD still alleviated airway remodeling through this pathway.

The surface receptors of ASMCs are activated to promote the process of airway remodeling through intracellular signal transduction and immune cell interaction. Herein, our in vitro experiments demonstrated that the expression levels of autophagy‐related proteins (i.e., the Beclin‐1 and LC3‐Ⅰ/LC3‐Ⅱ) were upregulated in ASMCs induced by the ATP/P2X7R axis, and the autophagy vacuoles were significantly increased based on the AO staining. The results indicated that the activation of the ATP/P2X7R axis aggravated autophagy, while PD blocked autophagy by inhibiting the ATP/P2X7R axis in ASMCs. Additionally, the ATP/P2X7R axis induced NLRP3 inflammasome activation in ASMCs, which not only upregulated the expression of ASC, NLRP3, and caspase‐1 but also promoted the secretion of IL‐1β after cleavage of IL‐1β by caspase‐1 activation. IL‐1β is one of the potent proinflammatory factors. The activation of the ATP/P2X7R axis induces ASMCs to secrete large amounts of IL‐1β. However, PD reversed the ATP/P2X7R axis and inhibited IL‐1β secretion. These results suggest that PD inhibited NLRP3 inflammasome and alleviated airway remodeling by regulating the ATP/P2X7R axis.

To further verify the relationship of the ATP/P2X7R‐NLRP3 axis with autophagy in airway remodeling, the ATP‐decomposing enzyme and inhibitor were applied. Our findings indicate that the ATP/P2X7R axis is involved in autophagy in the airway remodeling of asthma, and autophagy may affect the activation of P2X7R–NLRP3. Caspase‐1 inhibitor pretreatment decreased the activation of IL‐1β in the absence of caspase‐1, which further confirmed the important role of NLRP3 inflammasome in airway remodeling. However, pretreatment of ASMCs with PD effectively inhibited the regulation of the ATP/P2X7R–NLRP3 axis on pro‐inflammatory effect and reduced the autophagy of ASMCs. Moreover, taking into account the regulatory effect of AMPK/mTOR and ATP/P2X7R signaling by PD in asthma, we explored the role of P2X7R activation in the AMPK‐mediated airway remodeling. Interestingly, our results showed that the P2X7R gene knockdown with siRNA in ASMCs could restore the activity of AMPK, which further indicated the importance of the P2X7R signaling pathway in airway remodeling.

PD can promote autophagy and apoptosis of cancer cells [58, 59]. However, there is also a study reporting that PD alleviated excessive autophagy and apoptosis to improve β‐cell survival [11]. As an anticancer drug, PD has been shown to exert strong anti‐inflammatory effects in recent years [60, 61]. Herein, we demonstrated that asthma could be alleviated by PD. One novel finding of this study is that PD may alleviate asthmatic airway remodeling by inhibiting excessive autophagy. In the future, we will further explore the relationship between PD and autophagy in other allergic diseases, including allergic rhinitis and allergic dermatitis. Another novelty of our study is that it specifically investigates the therapeutic potential of PD in modulating the ATP–P2X7R–NLRP3 inflammasome axis and the LKB1/AMPK/mTOR autophagy signaling pathway. Unlike the study by Zeng et al. [29], which primarily examined PD's effects through promoting Nrf2‐mediated antioxidation to alleviate epithelial–mesenchymal transition and fibrosis in asthmatic airway remodeling, we provide novel insights into the interplay between cellular signaling mechanisms and their role in influencing airway remodeling. By elucidating these mechanisms, our findings advance the current understanding of the potential therapeutic applications of PD in managing asthma, particularly concerning airway remodeling.

This study has some limitations. For example, while PD is known to modulate both autophagy and apoptosis, our study specifically focused on its role in suppressing pathological autophagy in asthma. In future work, we will compare the effects of PD on autophagy and apoptosis in asthma and explore crosstalk between autophagy and apoptosis pathways. Moreover, while our study provides insights into the therapeutic effects and mechanisms of PD in asthma, specific data on the lung distribution of PD and its pharmacokinetic profiles in various tissues are lacking. Future studies are essential to elucidate the pharmacokinetic parameters of PD, including its tissue distribution, half‐life, and metabolism, which will provide more comprehensive insights into its therapeutic potential in clinical settings. Addressing these pharmacokinetic aspects will be critical for translating our findings into clinical applications and understanding the full impact of PD on asthma and other respiratory conditions.

## 5 Conclusion

**Figure 9:**
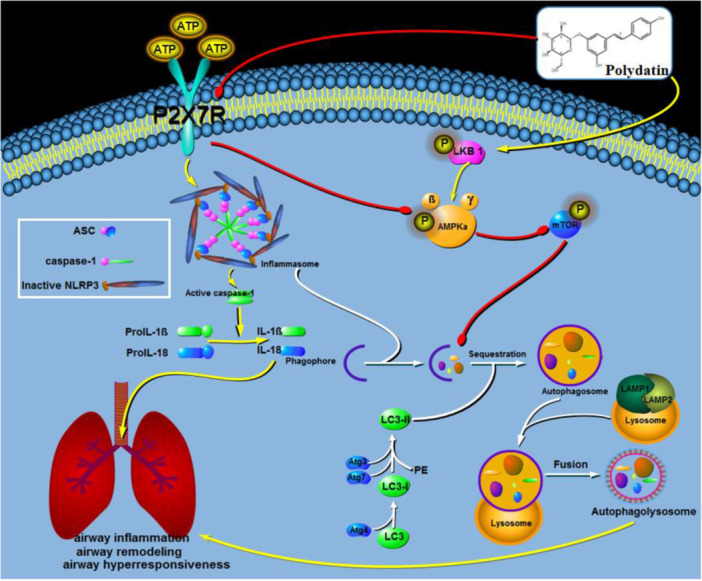
Schematic illustration of the mechanism of PD inhibiting ATP‐mediated airway smooth muscle cells. PD not only alleviates airway inflammation by regulating the LKB1/AMPK/mTOR signaling pathway but also regulates the NLRP3 inflammasome by inhibiting the ATP–P2X7R axis, which is involved in blocking the occurrence and development of autophagy and alleviating airway remodeling in asthma.

In conclusion, we demonstrated that PD inhibited the airway inflammation mediated by the ATP/P2X7R axis, decreased the production of mucus in lung tissue, reduced the airway responsiveness, and regulated the cytokines secreted by Th2, Th17, and Th22 cells, thus alleviating airway remodeling. We also found that PD alleviated airway remodeling via the LKB1/AMPK/mTOR signaling pathway. Moreover, PD inhibited excessive autophagy and NLRP3 inflammasome activation by regulating the ATP/P2X7 axis, reduced the expression of ASC, NLRP3, and caspase‐1, and decreased the secretion of IL‐1β and IL‐18 (Figure 9). Notably, while the BALB/c mouse model and OVA‐induced asthma are widely applied in asthma research due to their ability to mimic several aspects of allergic asthma, they might not fully reflect the complexity of the different endotypes of human asthma (such as neutrophilic and steroid‐resistant asthma). In subsequent research, we will employ models that more closely approximate the complexity of human endotypes to validate PD's efficacy against diverse inflammatory phenotypes, thereby providing direct evidence for clinical translation. Nevertheless, our findings on the role of PD in modulating the ATP/P2X7R–NLRP3 inflammasome–autophagy axis provide a foundation for further exploration into its potential clinical applications.

## Author Contributions

**Guangxing Li:** methodology, formal analysis, supervision, data curation, resources, writing – original draft, validation, software, conceptualization. **Liangchang Li:** conceptualization, methodology, investigation, funding acquisition, writing – original draft. **Zhiguang Wang:** conceptualization, writing – original draft, methodology, investigation, software, formal analysis. **Yihua Piao:** investigation, methodology, writing – review and editing. **Yilan Song:** writing – review and editing, methodology, investigation. **Li Li:** writing – original draft, resources. **Chang Xu:** writing – review and editing, software, formal analysis. **Xiaowan Li:** conceptualization, resources, funding acquisition, writing – review and editing, supervision. **Guanghai Yan:** conceptualization, writing – review and editing, funding acquisition, resources, project administration. All authors have read and approved the final manuscript.

## Ethics Statement

The experimental procedures were approved by the Ethics Committee of the Medical College of Yanbian University (Approval Number: JN.No20190515b068). The work described has been carried out in accordance with the relevant guidelines and regulations. All animal studies complied with the ARRIVE guidelines.

## Consent

The authors have nothing to report.

## Conflicts of Interest

The authors declare no conflicts of interest.

## Data Availability

The data underlying this article will be shared on reasonable request to the corresponding author.
